# Evolutionary dynamics of separate and combined exposure of *Pseudomonas fluorescens* SBW25 to antibiotics and bacteriophage

**DOI:** 10.1111/j.1752-4571.2012.00248.x

**Published:** 2012-02-23

**Authors:** Patricia Escobar-Páramo, Claire Gougat-Barbera, Michael E Hochberg

**Affiliations:** Institut des Sciences de l'Evolution, UMR5554, Université Montpellier IIMontpellier, France

**Keywords:** antibiotic resistance, bacteria, experimental evolution, hypermutator, phage therapy, *Pseudomonas*

## Abstract

The use of bacteriophages against pathogenic bacteria in health care and in the food industry is now being advocated as an alternative to the use of antibiotics. But what is the evolutionary response for a bacterial population if both antibiotics and phages are used in combination? We employ an experimental evolution approach to address these questions and exposed *Pseudomonas fluorescens* SBW25 and a related hypermutator strain (*mutS*−) to the action of the antibiotic rifampicin and the lytic bacteriophage SBW25ϕ2. We then compared the densities, growth rates, and the mutations at the *rpoB* locus leading to rifampicin resistance of the evolved bacterial populations. We observed that the evolutionary response of populations under different treatments varied depending on the order in which the antimicrobials were added and whether the bacterium was a hypermutator. We found that wild-type rifampicin-resistant populations involved in biofilm formation often reverted to rifampicin sensitivity when stresses were added sequentially. In contrast, when the mortality agents were added simultaneously, phage populations frequently went extinct and the bacteria evolved antibiotic resistance. However, populations of the hypermutator *mutS*− converged to a single genotype at the *rpoB* locus. Future investigation on other bacteria and using different antibiotics and bacteriophage are needed to evaluate the generality of our findings.

## Introduction

The widespread nature of antibiotic-resistant bacteria has become a considerable burden to human health and animal husbandry, and eliminating resistant genotypes from the environment is increasingly challenging. Suspending the use of particular antibiotics has proven ineffective in reducing resistance, because costly resistance mutations are compensated without losing resistance (Lenski 1998).

The use of bacteriophages to treat bacterial infections (so-called phage therapy) is now being advocated as an alternative to antibiotic therapies (e.g., Chanishvili et al. 2001; Levin and Bull 2004; Kutter et al. 2010). In phage therapy, lytic phages invade specific bacterial strains causing metabolic disruption and cell lysis. This selective agent, possibly together with an organism’s immune response, lowers the bacterial population to levels where it is no longer a danger to the organism (Kutter 2009). There are many examples of the successful treatment for bacterial infections in experimental and natural settings. For example, lethality of *Staphylococcus aureus*-induced infections in mice was successfully controlled by the addition of purified phage, such as ϕMR11 (Matsuzaki et al. 2003) or M^Sa^ (Capparelli et al. 2007). More recently, Hung et al. (2011) showed experimentally in a mouse model that the use of lytic phage ϕNK5 was highly effective in the treatment for *Klebsiella pneumoniae*-induced liver injuries, such as necroses and abscesses.

Whereas selection for antibiotic resistance creates a population of resistant strains that can persist in the environment once compensation for fitness has occurred (Levin et al. 2000), in phage therapy populations of bacteria and phages potentially antagonistically coevolve. Here, bacterial populations, if sufficiently genetically variable, will evolve resistance to phage attack. This, in turn, will select for novel phage genotypes that overcome the resistant phage genotypes (e.g., Levin and Bull 2004; Brockhurst et al. 2007). The relevance of coevolution for phage therapy is yet to be established (Payne et al. 2000; Cairns et al. 2009), but given evidence that some bacterial pathogens may coevolve with their phage pathogens (e.g., Mizoguchi et al. 2003), it is important to explore this potentially important mechanism in therapy research (Levin and Bull 2004).

One possible use of phages for bacterial control is in conjunction with antibiotics. Such combined therapies hold the promise of better control and the slowing of resistance evolution to antibiotics (Cairns and Payne 2009; Kutateladze and Adamia 2010). However, studies devoted to predict the appearance and evolution of resistant populations to both antimicrobial agents are necessary for their rational use when treating bacterial infections. Little is known about the evolutionary effects of both therapies on bacterial populations, either when applied in sequence or simultaneously. Sequential applications are important to understand, because, for example, the type of mechanism responsible for antibiotic resistance may condition the bacterial response to phage attack (Snyder 1972; Schwarz et al. 1981) and therefore the effectiveness of phage therapy. The simultaneous addition of both selective agents may result in the selection of bacterial variants capable of resisting this complex environment. Resistance may occur either by the use of multipurpose mechanisms or by the convergence to a single optimal variant capable of adapting to both stresses (Baquero et al. 1998). In such circumstances, bacterial population persistence will be more likely if large amounts of genetically based variation for adaptation are present, either through standing variation when stresses are introduced or mutations emerging thereafter (Bell and Collins 2008). More genetically variable populations will be associated with the presence of hypermutator bacterial strains, and study has shown that coevolution with phages may actually promote mutator emergence (Pal et al. 2007). Because hypermutator bacteria are abundant in nature, including those associated with infectious diseases (Matic et al. 1997; Denamur et al. 2002), the potential effect of hypermutator genotypes on the bacterial response to single or combined therapies needs to be addressed.

Experimental evolution represents a promising way to examine the evolutionary response of bacteria to the combined effects of antibiotics and phages. We experimentally investigate the population and evolutionary effects of the antibiotic rifampicin and the lytic phage SBW25ϕ2 on the gram-negative bacterium *Pseudomonas fluorescens* SBW25. A particular feature of the *P*. *fluorescens* SBW25 – SBW25ϕ2 phage interaction is that it can exhibit persistent antagonistic coevolution in laboratory culture (e.g., Buckling and Rainey 2002; Brockhurst et al. 2007; Poullain et al. 2008). *P. fluorescens* in the wild is mostly known as a phenazine producer and hence by its biological control properties that confer resistance to plant roots against parasitic fungi (Mavrodi et al. 2006; Escobar-Páramo et al. 2009). But it is also the most common microorganism isolated in spoiled raw or pasteurized milk, contaminated fresh meat products, and refrigerated foods, thus representing a considerable cost to the food industry (Arnaut-Rollier et al. 1999; Dogan and Boor 2003). *P.* *fluorescens* is also a useful model organism for experimental studies given its ability to form biofilms under natural and laboratory conditions (Rainey and Travisano 1998). The use of phages to control biofilm formation by certain strains of *P. fluorescens* has proven to be effective (Sillankorva et al. 2004). Interestingly, in certain cases, biofilm formation can be a specific defensive reaction to the presence of antibiotics (Hoffman et al. 2005), suggesting that both monitoring biofilm-forming cells and mutations conferring antibiotic resistance may be necessary to obtain a more complete assessment of overall antibiotic resistance.

We exposed *P. fluorescens* SBW25 populations to single or combined applications of rifampicin and the lytic phage SBW25ϕ2. We focused on two contrasting scenarios. In the first, we simulated cases where both control agents were applied simultaneously (‘simultaneous treatment’), with the aim of slowing or preventing resistance to one or both agents owing to their additive effects on the bacterial population. In the second, we simulated instances where resistance to the antibiotic was already present in the bacterial population at the time when phage was applied (‘sequential treatment’). To assess the potential effect of hypermutators in response to the antimicrobial agents, we also compared populations of wild-type *P*. *fluorescens* SBW25 [hereafter referred to as WT] with an isogenic hypermutator strain *mutS*− [hereafter referred to as *mutS−*]. This latter strain is a constructed SBW25 *mutS* knockout mutant that has a mutation rate of *c.* 10^−5^ per base pair, per generation (Pal et al. 2007) (the WT has a mutation rate of *c.* 5 × 10^−7^ per base pair, per generation). We therefore expected populations of *mutS−* to have higher initial genetic variation than WT populations in the simultaneous treatment and to be able to generate new mutations at a higher rate between the first (antibiotic) and second phases of selection (phage) in the sequential treatment.

Our results suggest that the evolutionary path in the adaptation of bacterial populations under the combined action of the antibiotics and the phages is difficult to predict and the response depends both on the order in which the antimicrobials are added and on the bacterial mutation rate. We discuss the implications of our *in vitro* study in the context of single and combined *in vivo* antibiotic therapies, and the unexpected result of the reversion of antibiotic resistance in biofilm-forming bacteria exposed to phages.

## Materials and methods

### Bacterial and phage strains

We used two strains of *Pseudomonas fluorescens* SBW25: the isogenic ancestor (wild type or ‘WT’) and a hypermutator (*mutS−*). The SBW25 *mutS−* knockout mutant was constructed by gene deletion and antibiotic marker recycling (for details, see Pal et al. 2007, Supporting Information). A population of the WT (Rainey and Bailey 1996) was grown in King’s B (KB) medium for 24 h at 28°C, under constant orbital shaking at 200 rpm. 20 μL aliquots of this culture were plated (after appropriate dilution) onto several KB-agar plates, and individual clones were then streaked on both KB plates and on KB plates supplemented with the antibiotic rifampicin at a concentration of 100 μg/mL. This concentration is lethal to individual bacterial cells not possessing a resistance mutation. Twelve susceptible and twelve resistant clones were arbitrarily selected from the KB (rif-sensitive) and the KB + rifampicin (rif-resistant) plates, respectively, inoculated into 6 mL KB liquid medium and incubated for 24 h at 28°C under constant orbital shaking at 200 rpm. These populations constituted the twelve rif-sensitive and twelve rif-resistant replicates. The same procedure was used to obtain the 12 rif-sensitive and 12 rif-resistant replicate populations of the isogenic SBW25 *mutS−* strain.

### Experimental regime

After 24 h of incubation, the selected replicate populations were used to start six experimental treatments for each strain (WT and *mutS−*). Thus, each treatment was replicated 12 times. Four of these treatments were started with rif-susceptible strains and the other two with the rif-resistant strains. In all, 144 microcosms were initiated by inoculating *c.*10^7^ bacterial cells into 2 mL of fresh medium in 24-well microtitre plates as follows: (i) Control: rif-susceptible bacterial cells were inoculated into and evolved in KB medium; (ii) Rifampicin: rif-susceptible bacterial cells were inoculated into and evolved in KB medium supplemented with (100 μg/mL) of rifampicin (‘KB+rif medium’); (iii) Phage: rif-susceptible bacteria and *c.*10^5^ particles of the lytic phage SBW25ϕ2 were inoculated and evolved in KB medium; (iv) Simultaneous: rif-susceptible bacteria and *c.*10^5^ phage particles were added simultaneously and evolved in KB+rif medium; (v) Sequential in KB medium: rif-resistant bacteria and *c.*10^5^ phage particles were added to and evolved in KB medium; (vi) Sequential KB+rif medium: rif-resistant bacteria and *c.*10^5^ phage particles were added to and evolved in KB+rif medium (this treatment simulated situations where selection for resistance to rifampicin was maintained after the addition of phage). Once started, none of the treatments experienced re-inoculations of either bacteria or phage.

The microcosms were incubated at 28°C and orbitally agitated at 200 rpm for 1 min every 30 min. Each culture was serially transferred every 2 or 3 days for a total of eight transfers (full sequence in days between transfers: 2-2-3-2-2-3-2-2, corresponding to about 50 bacterial generations). At each transfer, each culture was well-mixed by pipetting before transferring 20 μL into 2 mL of fresh medium (depending on treatment, either KB or KB+rif). At the end of the experiment, 20 μL of each culture was used to assay bacterial densities by counting colonies grown on KB-agar and KB-rif-agar plates. 150 μL samples of each population were frozen in 80% glycerol at −80°C. The presence or absence of phages at the end of the experiment in all phage treatments was determined by adding 10 μL of extracted aliquot on soft agar containing exponentially growing ancestor *P. fluorescens* SBW25, and the observation of plaques was used as evidence of phage presence.

### Molecular determination of genotypic diversity

Changes in genotypic diversity were determined by sequence analysis of cluster II (from nucleotide 1539 to 1737), corresponding to AA513 to AA579 of the gene coding for the β-subunit of RNA polymerase (*rpoB*) that is responsible for mutational resistance to rifampicin (Jin and Zhou 1996). This was performed by PCR amplification using the primers LAPS and LAPS27 (Tayeb et al. 2005). Only the first six of the 12 replicate populations per treatment were considered. A total of 78 sequences were analysed, including (i) the six rif-sensitive ancestor and six rif-resistant genotypes from the first step of the sequential treatment, both of WT and *mutS−*; (ii) one clone from each of WT replicate populations 1, 2, and 3 in both the control and the phage treatments; (iii) one clone from WT and *mutS−* replicate populations 1 to 6 of the following treatments: rifampicin, simultaneous, sequential in KB, and sequential in KB+rif.

At the end of the experiment, bacterial clones were arbitrarily chosen from the same KB-agar plates that were used previously to determine final population densities. DNA from these selected clones was extracted by boiling colonies at 100°C for 10 min. Sequences were aligned using the program ClustalW2 (Larkin et al. 2007). Sequences were deposited in GenBank under accession numbers: FJ834330–FJ834431.

### Post-treatment bacterial population growth in KB medium

We determined the growth of the ancestral clone and of 66 of the sequenced bacterial isolates from the treatments by incubating them in KB medium for 24 h at 28°C, under constant orbital shaking at 200 rpm. Population sizes were recorded as the mean optical density (OD) at 660nm of three replicates of each sample and population growth as log_10_(OD 24 h/OD initial). For logistical reasons, we did not conduct these assays on *mutS−* treatments confronted with phage.

### Statistical analyses

Bacterial population densities at the end of the experiment were compared across treatments. All analyses were performed as a single GLM anova with the following five factors: strain type, presence or absence of rifampicin, presence or absence of phages, and simultaneous or sequential addition of the stresses. Analyses on bacterial densities were conducted from log-transformed data. Comparisons of the growth rates of single selected clones from six populations of each treatment were performed on OD660 data by means of a separate one-way anova for each strain. All analyses were performed using JMP (1989–2007).

## Results

### Combining antibiotic and phage: effect on bacterial population density

**Figure 1:**
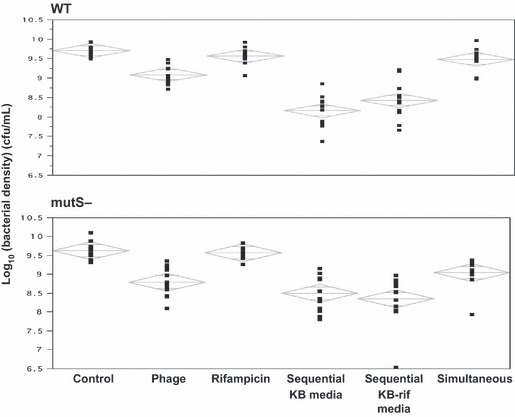
Log_10_ of bacterial densities (in cfu/mL) of the 12 replicate populations of *P. fluorescens* SBW25 wild type (WT) and the hypermutator (*mutS−*), estimated after eight serial transfers under the effects of rifampicin, the lytic bacteriophage SBW25ϕ2, or both. Simultaneous and sequential treatments refer to the temporal sequence in which rifampicin and phage were introduced. In the ‘sequential treatments,’ phages were added after the addition of rifampicin (and the concomitant selection of rif-resistant bacteria). The central line in each diamond is the group mean, the upper and lower lines within the diamonds represent the 95% overlap marks (i.e., how far the diamonds can overlap with the sample means still significantly different), and the upper and lower points of the diamonds represent the 95% confidence limits.

The effect of treatments on final population densities was similar in WT populations and *mutS−* populations (*F*_1,138_ = 1.96; *P* = 0.163). While selection with rifampicin did not have an effect on final bacterial density (*F*_1,138_ = 0.08; *P* = 0.77), as expected, the presence of phages significantly reduced both *P*. *fluorescens* SBW25 WT and the hypermutator *mutS−* populations (*F*_1,138_ = 44.78; *P* < 0.0001) (Table 1 and Fig. 1). This effect was even more prominent when the two stresses were applied sequentially (i.e., when phage was added to populations already resistant to rifampicin) (*F*_1,138_ = 31.14; *P* < 0.0001) (Table 1 and Fig. 1), suggesting a connection between antibiotic resistance and the ability to cope with phages. Furthermore, when the two stresses were applied simultaneously, WT populations attained high final densities (Fig. 1), but the phage population appeared to go extinct (i.e., was undetectable when plated on ancestral WT bacteria) in 7 of the 12 WT replicate populations (not shown). In contrast, *mutS−* populations persisted at lower densities and phages were present throughout the experiment (Fig. 1).

**Table 1:** anova for the effects on final bacterial densities of strain type, antimicrobial identity, and the order in which they were added

Analysis of variance					

Source	DF	Sum of squares	Mean square	*F* ratio	*P*
Model	5	212.3108	42.4622	55.4594	<0.0001
Error	138	105.6589	0.7656		
Total	143	317.9697			

Source	DF	Sum of squares	*F* ratio	*P*
Strain type	1	1.5063	1.9673	0.1630
Antibiotic	1	0.0648	0.0846	0.7716
Phage	1	34.2922	44.7887	<0.0001
Simultaneous	1	5.0667	6.6175	0.0112
Sequential	1	23.8454	31.1443	<0.0001

### Treatment effects on bacterial population growth

**Figure 2:**
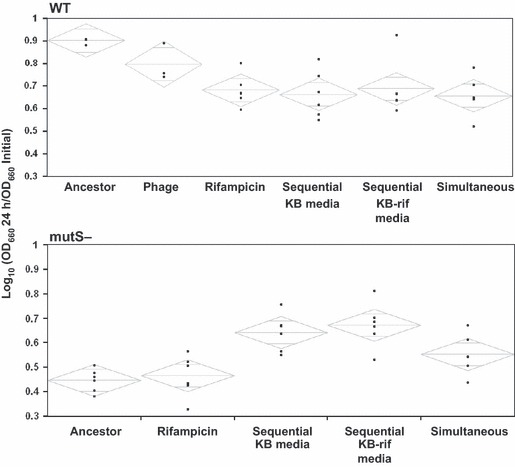
Treatment effects on bacterial growth measured as log_10_ (OD660 24 h/OD660 initial). Data are from 66 selected clones from the different experimental treatments. Diamonds drawn as in Fig. 1 based on transformed data. See main text for methods.

At the end of the eight serial transfers, populations were grown for an additional 24 h and their optical densities (OD660) measured. We observed that the growth of all treatment populations (Fig. 2) was below that of unevolved WT *P. fluorescencs* SBW25 (mean log_10_(OD 24 h/OD initial) = 1.09, SD = 0.10; data from assay in Figure S1), suggesting a cost of adaptation in terms of population growth. Among the treatments, we found that rif-resistant WT populations had reduced growth after 24 h of incubation compared to nonresistant ancestral strains (*F*_9,44_ = 6.68, *P* < 0.0001), and that *mutS−* ancestors had lower population growth than the WT ancestors (*F*_1,11_ = 549.52, *P* < 0.0001), suggesting that putative costs of evolving rifampicin resistance and the costs of the *mutS−* genetic construction are both expressed under these experimental conditions (Fig. 2A). But in contrast to the WT, evolved rif-resistant *mutS−* populations had similar, and in some cases even higher, growth than their ancestors (*F*_7,40_ = 5.07, *P* < 0.001; Fig. 2B), which indicates that compensation for the cost of rifampicin resistance may have occurred. An alternative explanation for this observation is that *mutS−* lines evolve faster, and more serial transfers would have been necessary for WT lines to adapt and reach higher population growth rates.

### Effect on mutations at the *rpoB* gene

We examined the genetic response of experimental populations by sequencing cluster II of the *rpoB* gene, encoding the subunit of RNA polymerase that is responsible for mutational resistance to rifampicin (Jin and Zhou 1996). Seven common mutations in this cluster were observed among the 78 sequenced bacterial isolates from the different populations (D521G, D521A, L526V, H531C, H531Y, L538P, and S579F; Table 2). All populations derived from rif-resistant ancestors maintained the ancestor’s mutation. The most common mutation among the rif-resistant WT and *mutS−* ancestors was D521G. When rifampicin and phage were added simultaneously, most WT populations showed the common mutation (D521G), but all *mutS−* populations converged to the uncommon genotype (D521A) at which aspartic acid (D) is replaced by Alanine (A) (Table 2).

**Table 2:** Amino acid substitutions in cluster II of the *rpoB* gene, responsible for mutational resistance to rifampicin, found in this study. The sequence at the top corresponds to the wild-type ancestral sequence of cluster II (from AA513 to AA579) of the *rpoB* gene of *Pseudomonas fluorescens* SBW25, and the amino acids in bold are those substituted in the resistant strains from this study. Note that *mutS−* populations evolving after the simultaneous addition of rif and phage in KB-rif medium (underlined) converged to a single genotype (D521A). *513 SSQLSQFMDQNNPLSEITHKRRVSALGPGGLTRERAGFEVRDVHPTHYGRVCPIETPEGPNIGLINS579*

WT	1	2	3	4	5	6
Sensitive ancestors	DLHLS	DLHLS	DLHLS	DLHLS	DLHLS	DLHLS
Rifampicin	DL**C**LS	**G**LHLS	D**VC**LS	DLHLS	DL**C**LS	DL**Y**LS
Phage	DLHLS	DLHLS	DLHLS	DLHLS	DLHLS	DLHLS
Simultaneous	**G**LHLS	DLHL**F**	**G**LHLS	DLHL**F**	**G**LHLS	**G**LHLS
*Sequential first selection*						
Rifampicin selection	DLH**P**S	**G**LHLS	**G**LHLS	DLHL**F**	**G**LHLS	DL**Y**LS
*Second selection*						
With phage in KB	DLH**P**S	**G**LHLS	**G**LHLS	DLHL**F**	**G**LHLS	DL**Y**LS
With phage in KB+rif	DLH**P**S	**G**LHLS	**G**LHLS	DLHL**F**	**G**LHLS	DL**Y**LS
*mutS−*						
Sensitive ancestors	DLHLS	DLHLS	DLHLS	DLHLS	DLHLS	DLHLS
Rifampicin	DLHL**F**	**A**LHLS	DLHL**F**	D**V**HLS	**A**LHLS	**G**LHLS
Phage	DLHLS	DLHLS	DLHLS	DLHLS	DLHLS	DLHLS
Simultaneous	**A**LHLS	**A**LHLS	**A**LHLS	**A**LHLS	**A**LHLS	**A**LHLS
*Sequential first selection*						
Rifampicin selection	**G**LHLS	**G**LHLS	**G**LHLS	**G**LHLS	DLH**P**S	**G**LHLS
*Second selection*						
With phage in KB	**G**LHLS	**G**LHLS	**G**LHLS	**G**LHLS	DLH**P**S	**G**LHLS
With phage in KB+rif	**G**LHLS	**G**LHLS	**G**LHLS	**G**LHLS	DLH**P**S	**G**LHLS

### Unexpected result: reversal to antibiotic sensitivity

**Figure 3:**
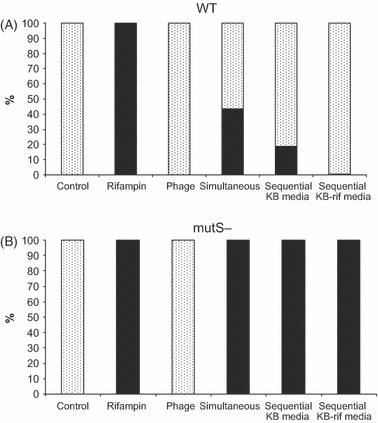
Relative frequencies of strains either resistant (black) or sensitive (dotted) to rifampicin estimated at the end of the experiment by counting bacterial colonies on KB plates containing rifampicin at a concentration of 100 μg/mL.

We plated the evolved populations on KB and KB+rif plates at the end of the experiment to estimate the frequency of resistant mutants. Surprisingly, all rif-resistant WT populations evolving in the presence of phage had more colony-forming units on KB plates than on KB+rif plates. Thus, in the five treatments where rifampicin and phage were added simultaneously and phages persisted, 56% of the bacterial cells were observed to be sensitive to rifampicin (Fig. 3A). This pattern was even more striking in the sequential treatments, where 81% of the bacteria became sensitive to the antibiotic when evolving in KB medium, compared to 99% when evolving in KB+rif medium (Fig. 3A). These results indicate that these populations showed some degree of reversal to rifampicin sensitivity. Our finding that the *rpoB* sequence of the newly sensitive strains was similar to that of the SBW25 WT ancestors suggests that back mutation is an unlikely explanation. Closer examination of the reverted WT strains revealed that they consistently exhibited a particular cellulose-producing, biofilm-forming morphotype—the wrinkly spreader (WS)—similar to that described by Rainey and Travisano (1998), but with a thicker cellulose layer (based on visual comparisons with WS produced by ancestral SBW25). This situation was not observed in the *mutS−* populations, which conserved antibiotic resistance even when evolving in the presence of phage (Fig. 3B).

## Discussion

Our results indicate that the evolutionary response of bacterial populations to the combined effects of antibiotics and phages depends on the order in which the antimicrobials are added. We found that in terms of the effects of single antimicrobial agents in reducing bacterial population size, phage SBWϕ2 has a larger effect than the antibiotic rifampicin. However, the combination of both antimicrobials is more effective in reducing bacterial density *in vitro* than either treatment separately. Because the mechanisms conferring resistance to phages and rifampicin may be different, the combination of phages and antibiotics diminishes the chances for developing resistance to both, resulting in enhanced control of resistant bacteria relative to single-agent treatments. But contrary to what some studies suggest for antibiotic therapy (D’Agata et al. 2008), we found that *in vitro*, the sequential addition of the antibiotic rifampicin and the phage SBW25ϕ2 was more effective at lowering *P. fluorescens* SBW25 populations than their simultaneous employment. We suggest that in the sequential treatment, the initial addition of rifampicin alters cluster II of the *rpoB* gene, leaving the population less able to evolve resistance to the phage.

The simultaneous addition of phages and antibiotics often drove the phage populations extinct and resulted in higher bacterial population levels by the end of the experiment. We suggest that the addition of both antimicrobials was sufficient to reduce WT populations below levels required for active phage replication (Payne et al. 2000; Cairns and Payne 2008), but that phage extinctions only occurred in microcosms in which few antibiotic-resistant cells were initially present. Once phage was lost, the antibiotic-resistant clones repopulated the experimental microcosms to high densities. Future studies on other microbial systems will be necessary to assess the generality of our results, which are based on single bacterial and phage species and strains.

### Importance of hypermutators to the evolutionary response

We found that under the simultaneous addition of antibiotic and phage, the hypermutator *mutS−* strains converged to the D521A allele (Table 2). All strains with D521A exhibited the resistant phenotype (Fig. 3B), increased size (Fig. 2B), and persisted with phage at high population densities (Fig. 1B). This suggests that this genotype may represent the best possible response to the two stresses, or that no other *rpoB* mutations occurred. Consistent with the former explanation, mutations at the *rpoB* gene are known to reduce bacterial fitness owing to pleiotropic effects on a variety of transcriptional and physiological processes (Jin and Gross 1989), including growth (Reid 1971), nitrogen metabolism (Ryu 1978), and the ability to support phage growth by altering the mechanism of phage replication within the host bacterium (Snyder 1972; Schwarz et al. 1981). The relationship between high mutation rate and ability to cope with phage has also been demonstrated, a possible mechanism being the hitchhiking of mutator alleles with those conferring (beneficial) resistance to phages (Pal et al. 2007).

Furthermore, we found that the high mutation rate of the *mutS−* strain favoured an actual increase in bacterial population growth when the antibiotic and the phage were added sequentially (Fig. 2B). This outcome is not surprising, because given enough time and genetic variation, bacterial populations are expected to reduce costs of resistance by fixing epistatic compensatory mutations instead of undergoing genetic reversal to sensitivity (Schrag et al. 1997; Perron et al. 2010). However, these populations were not protected from phage predation and, as such, the final population densities were significantly reduced (Fig. 1B). In contrast, WT populations (with lower mutation rates than the *mutS−*) were not able to reduce the cost of antibiotic resistance (Fig. 2A), and preferentially fixed the most common *rpoB* mutation D521G when confronted with the two selective pressures (Table 2). Interestingly, a significant fraction of these populations reverted to antibiotic sensitivity (Fig. 3A).

### An unexpected result: biofilm-forming WT rif-resistant strains reverted to a rif-sensitive phenotype in the presence of phages

How could these reverted strains persist in our experiment in the presence of the two stresses? It is known that in heterogeneous environments (e.g., static microcosms), *P. fluorescens* can generate the wrinkly spreader (WS) phenotype involved in biofilm formation (Rainey and Travisano 1998; Spiers et al. 2003). Bacteria in biofilms are capable of resisting phage predation and antibiotics (Stewart and Costerton 2001; Dunne 2002; Hall-Stoodley et al. 2004). In our experiment, the WS phenotype formed biofilms on the inner surfaces of the microcosms and would have been protected to some extent from phage predation and rifampicin mortality.

Reversal to antibiotic sensitivity has been reported in a variety of bacterial species. For example, in *Mycobaterium smegmatis*, genetic antagonism between mutations in the *rpoB* gene and *rpsL* genes conferring resistance to streptomycin resulted in the reversal of the mutated nucleotide to wild-type conditions with concomitant reversion to antibiotic sensitivity (Karunakaran and Davies 2000). In our study, both the WS phenotype and rifampicin resistance are metabolically costly, reducing bacterial growth in nonselective environments. We suggest that these two characters are likely to be negatively associated and exhibit additive costs, because the *rpoB* gene affects protein synthesis (Jin and Gross 1989), whereas the WS phenotype results from the modification of more than 30 kb of DNA encoding traits involved in the over-expression of cellulose-like protein production (4244). Thus, we propose that selection by phages for WS results in the reversion of antibiotic resistance, but this action is conditional on these reverted cells being protected from rifampicin selection in the D521A. The generality of this result remains to be evaluated with other antibiotics and other bacterial species.

Based on these findings, we suggest that employing phages to kill bacteria may have an as of yet unrecognized positive impact, by contributing to the reduction of antibiotic-resistant clones in cells dispersing from compromised biofilms (Hall-Stoodley et al. 2004). Differences in the mechanisms of antibiotic resistance may affect how strains overcome the combined effects of antibiotics and phages, particularly when resistance is coded on plasmids or other transferable elements.

Our results are of potential importance for antimicrobial therapies. We showed that for our experimental system, antibiotics followed by phages are a better strategy than their simultaneous employment for reducing bacterial populations, including those of widespread antibiotic-resistant clones and of mutator bacteria. In contrast, the simultaneous addition of both agents most likely results in the loss of phages followed by the establishment of an antibiotic-resistant population, or in the case of mutator bacteria, the emergence of a genotype that allows adaptation to both stresses.

*In vivo* antibiotic and phage therapies differ substantially from our *in vitro* experimental study in numerous ways, such as the length of therapy, the effect of host organism immune systems on bacterial populations, the genetic diversity of phages and of bacteria, and the morphotypic composition of the bacterial population (e.g., the presence of biofilms). Moreover, we only assessed the effects on population sizes, growth rates, and antibiotic resistance at the end of the experiment (i.e., after eight serial transfers). Future studies should track these variables during each transfer; this would be more akin to the dynamics of real antibiotic and phage therapies in hosts infected with pathogenic bacteria.

We argue that the use of experimental evolution opens important avenues of research for understanding the evolutionary consequences of phages on antibiotic-resistant bacteria. As shown in this study, the evolutionary response to the combined effects of antibiotic and phages is difficult to predict, depending to some extent on the order in which the antimicrobial agents are added, and on bacterial mutation rates. We conclude that these two factors condition the way genetic diversity is created and maintained in bacterial populations during the process of adaptation.

## Data archiving statement

Data deposited in the Dryad repository: doi:10.5061/dryad.4p49n3mj.

## Supporting Information

**Figure S1.** (A) *Pseudomonas fluorescens* SBW25 growth curve. (B) Correlation between OD660 and bacterial density in cfu/mL.
