# Supplementary figures and images for: Evolutionary dynamics of separate and combined exposure of Pseudomonas fluorescens SBW25 to antibiotics and bacteriophage
Source: Evol Appl. 2012 Feb 23;5(6):583–92. doi: 10.1111/j.1752-4571.2012.00248.x (PMC3461141; doi:10.1111/j.1752-4571.2012.00248.x)

A.

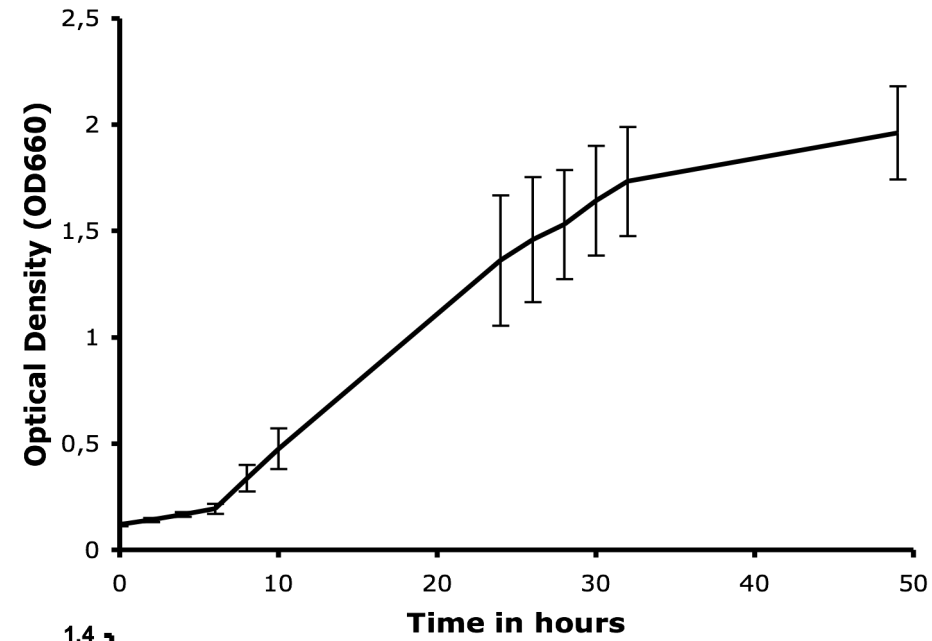

B.

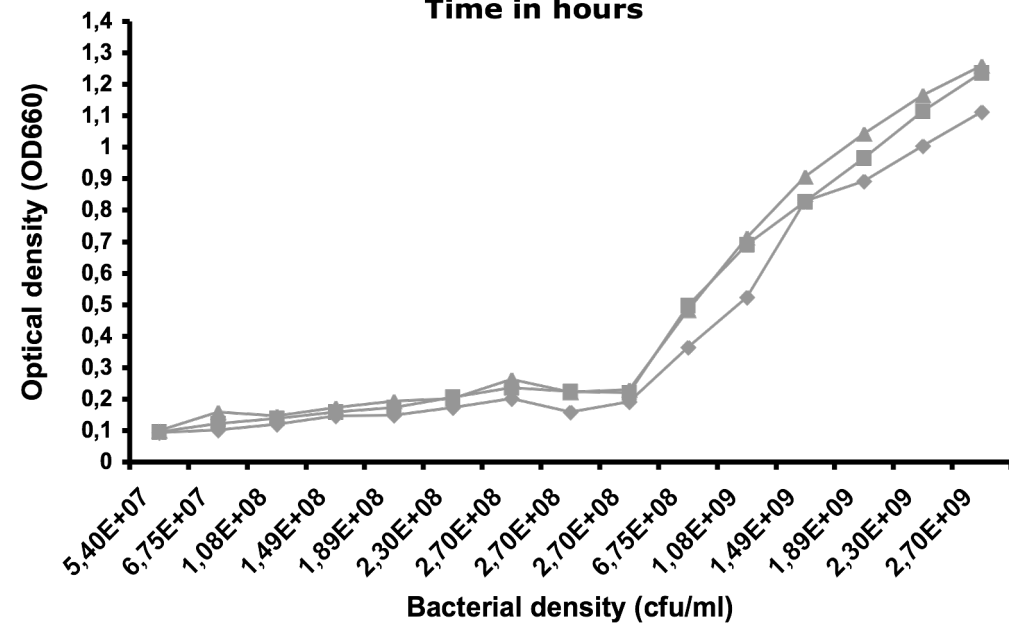

Supplement: Supplementary file 1 [file eva0005-0583-SD1.pdf]
