# Supplementary material for: Evolutionary dynamics of separate and combined exposure of Pseudomonas fluorescens SBW25 to antibiotics and bacteriophage
Source: Evol Appl. 2012 Feb 23;5(6):583–92. doi: 10.1111/j.1752-4571.2012.00248.x (PMC3461141; doi:10.1111/j.1752-4571.2012.00248.x)
Supplement: Supplementary file 2 [file eva0005-0583-SD2.doc]

**Supplementary Figure 1.**

**A.** ***Pseudomonas fluorescens* SBW25 growth curve.**

An isogenic culture of the reference strain of *P. fluorescens* SBW25 was inoculated into 6 ml of KB medium and incubated for 24 hours at 28°C under constant orbital agitation. Twenty microliters of this overnight culture was plated on KB-agar medium, and 24 individual colonies were selected from the plate. The colonies were resuspended in 6 ml of KB medium and incubated for 24 hours at 28°C under constant orbital agitation. New cultures were initiated by taking 20 μl of these new cultures and added into 180 μl of fresh KB liquid medium on a 96-well microtitre plate. For each colony, two replicate cultures were started. The microtitre plate was then incubated at 28°C in an orbital shaker for 49 hours. The OD660 of these initial cultures was measured at the beginning of the incubation time (T0) and after 2, 4, 6, 8, 10, 24, 26, 28, 30, 32, and 49 hours incubation.

**B. Correlation between OD660 and bacterial density in cfu/ml.**

The bacterial density of an overnight culture of the reference strain of *P. fluorescens* SBW25 was determined by plating 20 μl on a KB-agar plate and incubated at 28°C for 48 hours.

Colony numbers were counted, and the bacterial density was estimated as 2.70E+09 cfu/ml. Three aliquots of 200 μl of this culture were placed in the first well of each of three different 96-well microtitre plates. Three serial dilutions of these aliquots were achieved by adding 20 μl of the overnight culture to 180 μl of fresh KB medium. Further dilutions were made by taking 170, 140, 110, 80, 50, and 20 μl of the overnight culture and added to the wells with fresh KB medium to a final volume of 200 μl. All dilutions were made in triplicate in each of the three replicate plates. The OD660 of all samples in the three plates was measured three times. The lines in the figure represent the average OD660 values for each microtitre plate.
